# The Immune Response to Tumors as a Tool toward Immunotherapy

**DOI:** 10.1155/2011/894704

**Published:** 2011-12-05

**Authors:** F. Pandolfi, R. Cianci, D. Pagliari, F. Casciano, C. Bagalà, A. Astone, R. Landolfi, C. Barone

**Affiliations:** Department of Internal Medicine, Catholic University of the Sacred Heart, Rome, Italy

## Abstract

Until recently cancer medical therapy was limited to chemotherapy that could not differentiate cancer cells from normal cells. More recently with the remarkable mushroom of immunology, newer tools became available, resulting in the novel possibility to attack cancer with the specificity of the immune system. Herein we will review some of the recent achievement of immunotherapy in such aggressive cancers as melanoma, prostatic cancer, colorectal carcinoma, and hematologic malignancies. Immunotherapy of tumors has developed several techniques: immune cell transfer, vaccines, immunobiological molecules such as monoclonal antibodies that improve the immune responses to tumors. This can be achieved by blocking pathways limiting the immune response, such as CTLA-4 or Tregs. Immunotherapy may also use cytokines especially proinflammatory cytokines to enhance the activity of cytotoxic T cells (CTLs) derived from tumor infiltrating lymphocytes (TILs). The role of newly discovered cytokines remains to be investigated. Alternatively, an other mechanism consists in enhancing the expression of TAAs on tumor cells. Finally, monoclonal antibodies may be used to target oncogenes.

## 1. Different Antigenicity of Tumors

An important role of the immune system is to identify and eliminate tumors. Transformed cells of tumors express antigens that are not found on normal cells; these antigens are called tumor-associated antigens (TAAs). The immune system recognized these antigens as not self and mounts an immune response against tumor cells. However, tumors develop several mechanisms to escape immune recognition. For instance, when T cells interact with tumors, they may deliver several potential inhibitory signals, including lack of proper costimulatory activity by tumor cells and induction of immunosuppressive Tregs [1, 2].

In the recent years, specific antigenic characterization has permitted us to study an increasing number of tumors, in particular regarding their ability to escape from immune response and to downmodulate TAA expression and secreting inhibitory molecules. This has resulted in the identification of tumors that elicit different immune responses: (1) strong immunogenic tumors, such as melanoma and renal cell carcinoma, (2) the majority of tumors, however, are poorly immunogenic tumors: these include, for instance, colorectal cancer, hepatocellular carcinoma, pancreatic carcinoma, prostate carcinoma, lymphomas and leukaemias, and others [3, 4].

The tumor-associated antigens (TAAs) expressed by tumors have several sources.

- Some are derived from oncogenic viruses like human papillomavirus, which causes cervical cancer [5]. The HPV oncoproteins E6 and E7 have crucial roles in various steps of carcinogenesis, inducing degradation of p53 and destabilization of pRb. Several clinical trials show that recombinant HPV vaccines are safe and effective in preventing persistent infection of HPV and associated anogenital lesions. Thus, prophylactic HPV vaccination may be an ideal preventive method for other HPV-associated cancers. Therefore, vaccine against papillomavirus may be considered a very effective antitumor agent [6–8].
- Other TAAs are cellular proteins usually present in the human body that are overexpressed or aberrantly expressed in tumor cells; furthermore, others TAAs are also products of mutated genes.
- In addition, TAAs may also be the products of oncogenes or mutated oncosoppressors.

The most useful response of the immune system against tumors is to kill the abnormal cells using CTLs, which abound among TILs [9, 10]. TAAs are presented on MHC class I molecules. This allows CTLs to recognize the tumor cell as abnormal. NK cells also kill tumor cells by cytotoxicity, especially if the tumor cells have fewer MHC class I molecules on their surface than normal; this being a common phenomenon in tumors. 

Upon activation, CTLs express on their surface the death activator designated Fas ligand (FasL) and the engagement of Fas/FasL pathway lead to mediated apoptosis of cancer cells [11, 12]. 

Despite the activity of the immune system, clearly, tumors may evade the immune system and become clinically evident. Tumor cells often have a reduced number of MHC class I molecules on their surface, thus avoiding detection by killer T cells. 

An important challenge in cancer immunotherapy is the identification of effective strategies for enhancing its clinical efficacy. One approach is based on adjuvants, capable of breaking tolerance against TAAs. Interferons-alpha (IFN-alpha) are pleiotropic cytokines belonging to type I IFNs, extensively used in the treatment of patients with some types of cancer and viral disease. IFN-alpha can increase the expression of surface antigens enhancing the immune response, acting as an effective adjuvant in cancer immunotherapy [13, 14]. In melanoma it has been demonstrated that IFN-alpha increases the accumulation of gp100-specific, IFN-gamma-secreting CD8+ T cells in the tumor, demonstrating its efficacy as an adjuvant for peptide vaccination and giving insight into its mechanism of action. This provides a rationale for clinical trials in which vaccination is combined with IFN-alpha therapy for melanoma [15]. In addition, IFN-alpha can promote the differentiation and activity of host immune cells. Notably, a special interest is currently focused on the use of dendritic cells (DCs) generated in the presence of IFN-alpha (IFN-DC) for the preparation of anticancer vaccines. An additional approach for enhancing the response to immunotherapy relies on its combination with chemotherapy [16].

Here we will briefly discuss the immunobiology of tumors. Because the topic is too vast for this paper, we will discuss two tumors: melanoma as an example of strong immunogenic tumor and colorectal cancer as an example for poorly immunogenic tumors.

### 1.1. A Strong Immunogenic Tumor: Melanoma

Malignant melanoma is one of the most aggressive malignancies in human and is responsible for almost 60% of lethal skin tumors. Therapy with IFN-alpha 2b, the only agent approved in the USA for adjuvant use in high-risk melanoma patients, has not shown consistent overall survival benefit in randomized trials and is associated with considerable toxicity. Melanoma is one of the first tumors that have been associated to the presence of local cellular inflammation. The description of a lymphocytic infiltration of primary cutaneous melanoma confirmed Virchow's suggestion of a direct connection between inflammation and cancer. The past 30 years have accumulated considerable evidence that many tumors elicit a significant immune response, and a more favourable prognosis is correlated with the levels of TILs [17]. Nevertheless, although tumor microenvironment TILs include tumor-reactive T cells, melanoma can escape the immune system and continue to grow and metastasize [1]. Studying these mechanisms of immune escape of the tumor will improve the strategies to overcome obstacles to successful immunotherapy of tumors.

Melanoma is characterized by the expression of several TAAs, which can be recognized by T cells, resulting in a strong immunological response to the tumor. These TAAs include gp100, Melan-A/Mart-1, tyrosinase, MAGE-A1, and NY-ESO [1, 18]. 

Recent data have demonstrated that combined therapeutical approach with chemotherapy and cancer vaccines may have positive effects in the treatment of advanced and metastatic tumors. Chemotherapy, alone or with the association of cancer vaccine, can improve the expression of TAAs and induce enhancement of the cancer-reactive CD8+ cytotoxic T cells (CTLs) [16]. Specific topoisomerase inhibitors can augment melanoma antigens production, suggesting that a combination of chemotherapy and immunotherapy may be of potential value in the treatment of otherwise insensitive cancers [19].

Moreover, recent data have demonstrated the implication of Tregs in the pathogenesis and in the progression of tumors. Tregs mediate their immunosuppressive action also by the expression of the negative costimulatory receptor CTLA-4.

Furthermore, in the last years, the identification of, somatic mutations in the gene encoding the serine-threonine protein kinase B-RAF (BRAF) in the majority of melanomas has resulted in an opportunity to test oncogene-targeted therapy for this disease. Patients with advanced metastatic melanoma have been treated with PLX4032 (Plexxikon; RG7204, Roche Pharmaceuticals), a potent inhibitor of BRAF with the V600E mutation; this treatment resulted in complete or partial tumor regression in the majority of patients [20, 21]. 

Melanomas share initiating genetic alterations such as oncogenic mutations in BRAF and NRAS and often show recurrent patterns of chromosomal aberrations. Alteration of cell cycle proteins (e.g., cyclin D1, pRb, and p16) has a role in transformation and progression in melanocytic tumors. Higher expression of PAR-1 (protease-activated receptor-1) is seen in melanoma cell lines and tissue specimens [22]. Upregulation of PAR-1 mediates high levels of Cx-43 (gapjunctional intracellular communication molecule connexin) expression. This molecule is involved in tumor cell diapedesis and attachment to endothelial cells [23]. Protein kinase C (PKC) mediates signals for cell growth and is a target of tumor-promoting phorbol esters in malignant transformation [24]. Downregulation of E-cadherin and upregulation of N-cadherin may be seen in melanoma cells. Such shift of cadherin profile may have a role in uncontrolled proliferation, invasion, and migration. Other studies demonstrated the association of vascular endothelial growth factor (VEGF) and VEGF-receptor family with progression and melanoma metastasis [22].

Effective cancer immunotherapy is dependent on the presence of large number of antitumor lymphocytes with appropriate homing and effector functions that enable them to seek out and destroy cancer cells *in vivo*. Adoptive cell therapy (ACT) refers to an immunotherapy approach in which antitumor lymphocytes are identified and grown *ex vivo* and then infused into the cancer patients, often along with vaccines or growth factors that can augment the *in vivo* impact of the transferred cells. ACT with autologous tumor infiltrating may mediate durable complete responses in patients with metastatic melanoma [25].

### 1.2. A Poorly Immunogenic Tumor: Colorectal Cancer (CRC)

Colorectal cancer (CRC) is one of the leading causes of death in the Western world. Immunotherapy could play a crucial role in patients with advanced disease at presentation permitting tumor regression or possibly clearance. Despite advances in research and treatment modalities, CRC still accounts for around half a million deaths yearly worldwide. Traditional and even newer pharmaceutical therapeutic regimens are limited in terms of tolerance, efficacy, and cross-resistance. Additional non-cross-resistant therapies with nonoverlapping toxicities are needed to improve the outcome for patients with CRC. Cancer vaccines, designed to activate immune effectors (T cells and antibodies) to prevent recurrence or treat advanced cancers, have now demonstrated clinical benefit [26].

Bonertz et al. have found that in CRC Tregs T-cells response is addressed against a limited repertoire of TAAs, which include p53, carcinoembryonic antigen (CEA), Her2/neu, and heparanase pp1 [27].

Colorectal tumor cells frequently express CEA which correlates with the state of the tumor, augmenting its expression in advanced phases. CEA is considered a clinical marker of this tumor, with utility in the diagnosis, prognosis and followup of the disease [28, 29]. Some authors have used anti-CEA antibodies tagged with radioactive Yttrium-90 [30] against CEA-expressing metastatic malignances or combined with antivascular antigens, like combretastatin and bevacizumab (anti-VEGF), or with gemcitabine [31–33]. Moreover, in recent years it has been shown that CRC can express other antigens, such as extracellular surface marker CD55 [34] and the oncofetal antigen 5T4. This latter is a surface glycoprotein expressed on a variety of human adenocarcinomas, including CRC, and plays an important role in tumor progression and metastasis. The expression patterns and functional role in the metastatic process suggest that 5T4 is a good target for vaccine development. A modified vaccine virus Ankara (MVA) encoding human 5T4 (designated TroVax) demonstrated therapeutic effects in murine tumor models and human T cells recognized 5T4 epitopes in an HLA-restricted manner. TroVax vaccine has been evaluated in clinical trials targeting patients with colorectal cancer of advanced stage (IV stage), renal cell carcinoma, and hormone refractory prostate cancer [35]. Results from clinical trials on metastatic colorectal cancer demonstrate that MVA-5T4 is safe and immunogenic as a monotherapy and in combination with standard-of-care therapies. MVA-5T4 induced potent and sustained immune responses in approximately 95% of tested patients. With its minimal side effects and ability to produce immune responses, MVA-5T4 is a promising addition to cancer therapy [36]. Moreover, preliminary results showed significant associations between 5T4-antibody responses and overall survival in patients with CRC. The 5T4-specific antibodies were present at higher levels in cancer patients compared with healthy donors and increased significantly after treatment with MVA-5T4 [37].

Furthermore, CRC can express oncogenes; in particular, KRAS mutations occur in almost 40% of CRC patients. KRAS is a cellular signalling effector downstream from the EGF/EGFR pathway. KRAS mutations are common in colorectal, ovarian, and lung adenocarcinomas. There have been recent attempts to quantify KRAS mutation and predict responses to treatment using an EGFR inhibitors (cetuximab) [38]. 

In addition, studying on TILs has permitted us to differentiate between the immune cellular populations: Tregs and Th17 cells are involved in the pathogenesis and the progression and proliferation of CRC malignant cells. In particular Tregs and Th17 cells are correlated with a poor prognosis of CRC and with advanced tumors [39]. According to their immune inhibitory function, Tregs depletion results in stronger TAA-specific immune response [27].

## 2. Biology of the Immune Response to Tumors

### 2.1. Immune Pathways That Can Potentially Limit Tumor Expansion

#### 2.1.1. Mechanisms of Action in Tumor Vaccines: DCs, CTLs, and Humoral Response

The discovery of high number of tumor-infiltrating lymphocytes (TILs) with skewed tumor-specific TCR expression has promoted the development of both adoptive immunotherapy with transfer of TAA-primed TILs into patients and vaccine-based antitumor therapy [1, 40]. CTLs mediate tumor destruction by the release of perforin [41] and granzymes or by the activation of the Fas-/FasL- mediated apoptosis [42].

Therapeutic tumor vaccines have two main objectives: priming Ag-specific T cells and reprogramming memory T-cells (i.e., a transformation from one type of immunity to another, e.g., regulatory to cytotoxic). Dendritic cells are essential in the generation of immune responses, and as such represent targets and vectors for vaccination [43]. The main goal of tumor vaccine therapy is the production of mature dendritic cells (DCs, the most specialized APCs) able to stimulate an antigen-specific T-cells response *in vivo *[44]. In classical protocol DCs are activated and loaded with TAAs or transfected with RNA-encoding tumoral epitopes and then transferred to tumor-bearing hosts [45, 46]. Notably, most antigens expressed on tumor cells are self antigens and may result in poor antigenicity due to negative selection of high-avidity autoreactive T-cell subsets; moreover antigens expression depend on the proteolytic processing by immunoproteasomes and differential binding to allelic MHC variants leading to the hiding of “cryptic” specific epitopes [47]. Therefore the antigen presentation may be different in different cells, and a selection of proper antigenic peptides may be useful to mediate efficient killing of cancer cells [47].

### 2.2. Immunological Pathways That Can Limit the Immune Responses

#### 2.2.1. The Role of Tregs

The heterogeneity of CD4 cells has been described in the past [48], but only recently a CD4 T-cell subpopulation with regulatory function (Tregs) has been characterized functionally. Regulatory T cells (Tregs) represent about 5–10% of peripheral CD4+ T cells; they are characterized by the ability to suppress T-cell responses. If this function is impaired, the host will be exposed to dysfunctions in self-tolerance. Several diseases have been linked to defective Treg activity including type I diabetes, allergy, and other autoimmune diseases [39, 49–51].

In tumors, several studies suggested a direct correlation between adverse prognosis and presence of Tregs in peripheral blood as well in TILs and in draining lymph nodes of different tumors [52].

Tregs express a number of chemokine receptors such as CCR2, CCR4, CCR5, CCR7, CCR8, and CXCR4 and are able to migrate in response to a variety of chemokines such as CCL22, CCL17, CCL1, and CCL4 [53–55]. Tregs may be recruits to the tumor site by the chemokine CCL22 produced by the tumor cells and tumor-associated macrophages (TAMs). Tregs accumulated via CCL2-CCR4 recognize tumor-associated immunogenic self-antigens (self-Ags) and proliferate [52]. Moreover, a recent study on breast cancer showed that the hypoxia environment drives the Tregs recruitment through both CXCL12 production by tumor cells and hypoxia-induced CXCR4 expression in Tregs [56].

Moreover, Tregs selectively recruited within the tumor site will be activated by mature DCs likely through TAA; therefore, Tregs induce T-cell suppressions in an antigen-selective manner [52]. Indeed it is also clear that vaccination with some of these epitopes, administered with or without an adjuvant or presented by *ex vivo* cultured antigen-presenting cells (APCs), can induce humoral and CTL antitumor responses in some cases [57].

It has been reported that immunosuppressive factors produced in the growing tumor environment, such as TGF-beta, IL-6, and IL-10, created an immunosuppressive environment. Therefore, both the tumor cells (by their expression of tumor antigens and production of these factors) and/or the TAMs may act via promoting the antigen-activated T cells to differentiate and proliferate into Treg cells [58].

Tregs suppression may therefore impair cancer immunotherapies [52]. Therefore a clear understanding of the mechanisms of action by Tregs in tumor immunity is needed to establish a useful tumor vaccine or immunotherapy [59, 60]. Tregs are highly specific for antigens, suggesting that they exert T-cell suppression in an antigen-selective manner [27].

Recent data, confirming the high presence of Tregs within TILs in the tumor site and in the tumor-draining lymph nodes, however, has demonstrated that regulatory T cells in TILs donot originate by conversion of T-conventional cells (T-conv). Tregs arise from different populations with unique TCR repertoires. Enrichment of Tregs within TILs most likely, therefore, reflects differences in the way that Treg and T-conv cells are influenced by the tumor microenvironment. Elucidating the nature of these influences may indicate how the balance between tumor-infiltrating Treg and T-conv cells can be manipulated for therapeutic purposes [61].

#### 2.2.2. The Role of CTLA-4 and PD-1

T-cell activation and inhibition are regulated by signalling of several molecules including CD28 that provides costimulation, CTLA-4 (CD152) that binds to the same ligands as CD28, but has more affinity and delivers an inhibitory signal, and programmed death-1 (PD-1) that may be involved in tumor evasion. All these molecules have a potential role in immunotherapy [39].

Remarkably, CTLA-4 has more affinity than CD28 for its ligands and can trigger T-cell anergy. CTLA-4 delivers inhibitory signals to T cells blocking their effector functions through different mechanisms including diminishing of TCR signalling, blocking cell cycle progression, and reducing IL-2 production [39].

Also PD-1 seems to be involved in immune evasion, and its expression is reported in melanoma TILs contributing to their impaired antitumor responses [62].

#### 2.2.3. The Role of Cytokines in Regulation of Tumor Antigens

Tumors can mediate their ability to escape immune recognition also secreting immunosuppressive cytokines, such as IL-10 and TGF-beta [63]. Furthermore Tregs can downmodulate immune response by cytokine secretion; these include IL-10, TGF-beta, and the discovered novel IL-35 [64–68]. IL-35 has been shown to be constitutively expressed by regulatory T cells and contributes to their suppressive activity. IL-35 is an important mediator inducing CD4+CD25+ T-cell proliferation and IL-10 production [69].

Recent data have demonstrated also a relation between cytokines and vitamins. In particular, vitamins A, D, and E modulate Treg function and IL-10 and TGF-beta production, involving the immune response mechanisms [70].

Moreover, in addition to the immune cells, also tumors can directly secrete immunosuppressive cytokines, further permitting them to evade the immune response. For example melanoma secretes oncostatin M (OSM), which transmits its signal via the gp130 cell surface receptor, resulting in the selective downmodulation of the melanocyte lineage antigens: Melan-A/MART-1, gp100, tyrosinase, tyrosinase-related proteins 1 and 2, and the M isoform of microphthalmia transcription factor [71]. On the other side it is important to underline that TAAs expression can be modulated in both directions. IFN-beta is an additional stimulus to TAAs expression in melanoma, including Melan-A/MART-1, gp100, and MAGE-A1, permitting an improve of immune response to melanoma cells [1, 18].

#### 2.2.4. The Role of Heat Shock Protein 90 (HSP90)

In recent years some data have revealed that the molecular chaperone Heat Shock protein 90 (HSP90) is involved in several condition, including cancer. Hsp90 regulates the trafficking of proteins in the cell, under stressful conditions, stabilizes its client proteins, and provides protection to the cell against cellular stressors such as in cancer cells. Through its role in regulating the conformation, stability, and function of several key, oncogenic client proteins, HSP90 contributes in maintaining malignant transformation and in increasing the survival, growth, and invasive potential of cancer cells. 

HSP90-inhibitors, such as geldanamycin and its analogue 17-allylamino-17-demethoxygeldanamycin (17-AAG, tanespimycin), determine suppression of MAPK pathway in malignant cells and may become new anticancer agents [72, 73]. Moreover, Banerji et al. have shown a correlation between oncogenic BRAF and NRAS mutations, frequently associated with malignant melanoma, and the HSP90. In fact NRAS mutations are stabilized by the molecular chaperone HSP90 and they are depleted by the HSP90 inhibitor 17-AAG [74]. In addition inhibitors may also upregulate TAAs [75].

## 3. Clinical Approach to Immunotherapy of Cancer

The increased understanding of the mechanisms of immunoregulation has suggested new strategies to design more effective cancer immunotherapies.

### 3.1. Vaccines: Prostate Cancer and Melanoma

Cancer vaccination is a kind of immunotherapy that relies on specific priming of the immune system in order to stimulate principally adaptive immunity against vaccine component, in contrast to nonspecific immunotherapy where the administered agent tries to enhance the innate immunity (e.g., Bacille Calmette Guérin). Early attempts to develop effective cancer vaccines had limited success due to the failure to identificate suitable target antigens, to mitigate the immunosuppressive environment and generate an effective immune response [76]. However, an improvement in our understanding of the immune system and tumor immunity, in particular, has facilitated the development of more promising vaccine strategies [77, 78].

Different vaccination strategies have been investigated including the use of whole-tumor cells or lysates, dendritic cells, peptide-base approach, recombinant proteins, and viral and DNA delivery vectors. Since antigens are poorly immunogenic by themselves, vaccines generally require the inclusion of potent immunoadjuvants to induce antitumor responses and a delivery system to effectively present the antigen to the immune system [77].

Sipuleucel-T represents the first cancer vaccine approved by the US Food and Drug administration for the treatment of metastatic hormone-refractory prostate cancer. Sipuleucel-T consists of autologous peripheral-blood mononuclear cells including antigen-presenting cells (APCs) that have been activated *ex vivo* with a recombinant fusion protein (PA2024) which contains prostatic acid phosphatase fused to granulocyte-macrophage colony-stimulating factor [79]. In a double-blind, placebo-controlled, multicenter phase III trial, patients with metastatic castration-resistant prostate cancer who received Sipuleucel-T had a prolonged overall survival (median survival 25.8 months in the sipuleucel-T arm versus 21.7 in the placebo group) showing a relative reduction of 22% in the risk of death as compared with placebo arm; also the rate of 3-year survival was increased for patients receiving Sipuleucel-T (31.7%) as compared with those receiving placebo (23%). In particular, patient in the Sipuleucel-T group who had an antibody titer of more than 400 against PA2024 or prostatic acid phosphatase at any time after baseline lived longer than did those who had an antibody titer of 400 or less (*P* < 0.001 and *P* = 0.08, resp.). Adverse events that were more frequently reported in the Sipuleucel-T group included chills, fever, and headache [79].

Several vaccines against melanoma antigens were tested in early clinical trials demonstrating a clinical benefit, but when tested in prospective randomized trials for advance melanoma, they failed to improve progression-free or overall survival compared with chemotherapy. The first evidence of clinical benefit of vaccination for patients with metastatic melanoma came from a prospective randomized phase III trial, conducted with stage IV or locally advanced stage III cutaneous melanoma, HLA A0201+ patients, without brain metastases who received high-dose IL-2 (720.000 IU/kg/dose) as the control group and a gp100 peptide containing a modified 209-217 (210M) epitope + montanide ISA followed by high-dose IL-2 as the experimental arm [80]. The modified g209–217 peptide (referred to as g209-2M) presents a methionine replacing the natural threonine at position 2; it bounds to the HLA-A2 molecule with greater affinity than the unmodified peptide, and it was shown to have an increased ability to generate melanoma-reactive CTLs. Response rate was significantly improved in the experimental arm as compared with control group (22.1% versus 9.7% (*P* = 0.0223), and also progression-free survival favoured the gp100-immunized patients compared to those treated with IL2 alone (2.9 months versus 1.6 months, *P* = 0.01). Overall survival was longer in the experimental group, but the difference was not significant (17.6 versus 12.8 months, *P* = 0.0964). 

Other vaccines containing multiple tumor-associated antigens including MAGE proteins, MART-1/MelanA, and gp100 were tested in a phase I/II trial in patients with advanced melanoma with evidence of clinical activity and durable responses [81]. Also vaccines containing dendritic cells pulsed with melanoma-associated antigens or autologous lysates [82], or electroporated with mRNA encoding CD40 ligand, constitutively active toll-like receptor 4, and CD70, are under investigations in metastatic melanoma patients [83].

A vaccine containing a tumor-associated antigen such as MAGE-A3 was also tested in a phase II study for patients with non-small-cell lung cancer after complete resection with improvement in disease-free and overall survival; on the basis of these results, a phase III study with this vaccine was initiated in 2007 and is currently ongoing [84].

Other vaccines produced promising phase III data such as vitespen, an autologous adjuvant vaccine for patients at high risk of recurrence after nephrectomy for renal cell carcinoma [85] and Biovaxid, an idiotype vaccine for patients with follicular lymphoma in first complete remissions [86].

Human telomerase reverse transcriptase (hTERT), the rate-limiting subunit of the telomerase complex, is another attractive target for cancer vaccination since telomerase is highly expressed in almost all cancer forms, while the expression in normal tissues is restricted. Phase I/II trials in advanced pancreatic and pulmonary cancer patients after vaccination with GV1001, a 16-aminoacids-peptide of hTERT sequence, have demonstrated some specific and durable T-cell responses, associated to a prolonged survival, without clinically important toxicity [87, 88].

### 3.2. Biological Drugs and Their Combination with Cancer Chemotherapy

In contrast to conventional chemotherapy, immunotherapy of tumors has raised the hope of a more specific therapeutical approach in oncology. In fact, immunotherapy, targeting TAAs, has permitted to use novel more specific molecules in cancer therapy.

As discussed above, biological therapies can also stimulate the immune response against cancer. In addition, as we will see, biological therapy can interfere with tumor blood vessel formation therefore blocking its ability to develop.

In some conditions, biological agents may also be administered together with chemotherapy in order to prevent cancer cells from repairing the DNA damage induced by chemotherapy itself. Biological agents can be grouped in two main classes; both these classes have an increasing number of drugs of potential interest and a complete review of them will require a volume and it is beyond the scope of this paper. We therefore will mention those that appear more promising, having in mind that, by the time our paper will appear, several new products will be introduced in the clinical practice.

### 3.3. Monoclonal Antibodies (mAbs)

Ideal drugs would be antibodies against specific antigens on cancer cells that are not cross-reactive with those on normal tissues. mAbs achieve their therapeutic effect through various mechanisms. They can have direct effects in inducing apoptosis or programmed cell death; they can block growth factor receptors, effectively arresting proliferation of tumor cells; they can bring about antiidiotype antibody formation enhancing the patient's immune response [89, 90]. 

mAbs can be associated to other substance such as a chemotherapy drug, radioactive particle, or a toxin in order to selectively deliver them to a specific cancer cell.

The first monoclonal antibody to receive FDA approval was rituximab, an antibody directed to the CD20 antigen [89, 90]. CD20 is a transmembrane protein whose intracellular portion contains phosphorylation sequences for protein kinase C, calmodulin, and casein kinase 2. Rituximab is active against B-cell lymphoproliferative diseases [89] that are the large majority of lymphoproliferative diseases [91]. When rituximab cross-links CD20 antigen, an increase in intracellular calcium is observed. This increase appears to activate the SER family of tyrosine kinases, resulting in further phosphorylation of the CD20 inner cytoplasmic chain and also phospholipase C-gamma. At the same time there is an upregulation of C-myc and myb messenger ribonucleic acid, an increase in adhesion molecule expression, and an upregulation of MHC class II proteins. The ultimate result is caspase 3 activation, causing cell apoptosis. 

Results of studies with rituximab alone as first-line treatment of low-grade non-Hodgkin's lymphoma have been encouraging [92, 93], as well as inhibition of p38 kinase [94]. Rituximab has been also combined with conventional CHOP chemotherapy (cyclophosphamide, doxorubicin, vincristine, and prednisone) for patients with intermediate grade or diffuse large-cell non-Hodgkin's lymphoma [95, 96].

Alemtuzumab is a mAb targeted at the CD52 antigen, found on the surface of most chronic lymphocytic leukemia (CLL) cells. It is particularly efficient in chemotherapy-resistant B-CLL. Binding of alemtuzumab to CD52 on target cells may cause cell death by 3 different mechanisms: complement activation, antibody-dependent cellular cytotoxicity, and apoptosis [97, 98].

In addition, mAbs can be used to deliver a toxin, such as the RFB4(dsFv)-PE38 (BL22), a recombinant immunotoxin containing an anti-CD22 variable domain (Fv) fused to truncated pseudomonas exotoxin [99]. CD22 antigen is found on the surface of hairy cell leukemia (HCL) cells. To target relapsed/refractory HCL, immunotherapy has been developed using anti-CD25 and anti-CD22 recombinant immunotoxins, or rituximab alone or combined with purine analogs. BL22 is now in phase I and II testing of relapsed/refractory HCL, achieving 47–61% complete remissions, several of them ongoing after 9-10 years [100].

Hematological malignancies show a wide variety of surface markers as potential target for mAbs targeting [91, 101–103]. In comparison to hematological malignancies, solid tumors have fewer specific targets for mAbs that are not cross-reactive with antigens on normal tissues. 

In 2006 the FDA approved trastuzumab, the first monoclonal antibody for the treatment of a solid tumour, in HER2 overexpressing breast cancer [104]. Trastuzumab works in several ways: downregulation of HER2 receptor expression; inhibition of proliferation of human tumour cells that over-express HER2 protein; enhancing immune recruitment and antibody-dependent cell-mediated cytotoxicity (ADCC) against tumour cells that overexpress HER2 protein, and downregulation of angiogenesis factors. Trastuzumab also increases the effect of chemotherapy on breast cancer cells (on the average the response rate rises from 50% up to 85%), and it is currently used in combination with different chemotherapy regimens in metastatic disease, in adjuvant and neoadjuvant setting [105, 106].

It is important to point out that there are several evidences suggesting that blockade of signal transduction may not be the only mechanism of action of mAbs since a potential role of immunologic mechanisms in the therapeutic efficacy of ErbB-targeted mAbs (as opposed to TKI) has been reported. Among the variables known to play a role in the antitumor activity of TA-targeted mAbs, there is their ability to mediate lysis of tumor cells *in vitro* by NK cells, monocytes, and granulocytes in an ADCC way. The extent of lysis is in turn influenced by several variables, and they, or at least some of them, may contribute to the differential clinical response of patients treated with mAbs-based immunotherapy [107]. mAbs also bind complement, leading to direct cell toxicity, known as complement-dependent cytotoxicity (CDC). 

Cetuximab is mAb effective for treatment of advanced colon cancer in combination with 5-fluorouracil, oxaliplatin, or irinotecan chemotherapy. It is also useful in locally advanced head and neck squamous-cell carcinoma when combined with radiotherapy or in recurrent head and neck cancer, combined with platinum-based chemotherapy [108].

Several papers have reported that the activity of cetuximab, as well as of panitumumab, is related to their link to the epidermal growth factor (EGFR) which prevents cancer cells from growing. In particular, it has been shown that these mAbs are effective only in patients whose cancer has no mutation of K-RAS gene, the so-called wild-type sequence. The mutation can be detected in about 40% of patients. The K-RAS mutations keep the EGFR always active so that its pathway can no longer be stopped by simply blocking the receptor [109].

Other mAbs have the function to enhance T-cell activation by blocking CTLA-4, a major negative regulator of T-cell-mediated responses. As we discussed previously, CTLA-4 is a homolog of CD28 that functions as an inhibitory receptor for B7 costimulatory molecules expressed on mature APCs. Anti-CTLA-4 mAbs with a much greater affinity for CTLA-4 than B7 may provide a survival advantage compared to vaccines or chemotherapy alone [90]. On the basis of these preclinical data, clinical trials have been initiated with two fully human anti-CTLA-4 mAbs, with different pharmacokinetic and pharmacodynamic profiles. 

Anti-CTLA4 blocking antibodies [110] are effective in the treatment of malignant melanoma and may increase Th17 cells in peripheral blood of patients with metastatic melanoma. However, anti-CTLA-4 antibody therapy is associated with autoimmune toxicity, due to the augmented cellular proinflammatory activity, as consequence of the increase of Th17 cells and of the inhibition of Tregs function [17]. 

Tremelimumab is a human IgG2 anti-CTLA-4 mAb with a serum half-life of approximately 22 days, the same reported for endogenous human IgG2, which is currently under evaluation at escalating doses (from 3 to 15 mg/kg every three months) in several phase I studies in patients with metastatic tumors such as pancreatic, breast [111] and renal cell carcinoma in combination with conventional therapies. As a single agent, tremelimumab did not demonstrate a clinically significant activity in a phase II study of patients with refractory metastatic CRC [112]; it generated durable tumor responses in a phase I/II trial of patients with treated metastatic melanoma [113], but it failed to produce a survival advantage in a randomized phase III study compared to conventional chemotherapy with dacarbazine or temozolomide [20].

Ipilimumab is a fully human monoclonal antibody IgG1 with a shorter half-life, which was tested at a dose of 3 mg/kg with or without dacarbazine and at 10 mg/kg as monotherapy every 3 weeks in several phase II studies in metastatic melanoma patients showing a significant activity with durable remissions [114]. These results have been confirmed recently in the first randomized phase 3 trial [115] in patients with previously treated advanced melanoma with ipilimumab significantly prolonging median overall survival both as a single agent (10.1 months; *P* < 0.003) and combined with a gp100 vaccine (10.0 months; *P* < 0.001) compared with vaccine control (6.4 months). Even more noteworthy was the improvement in long-term survival at 24 months from 13.7% (gp100 alone) to 21.6% and 23.5% for the combination and single ipilimumab, respectively [115]. In addition some patients who progressed after an initial response (consisting of stable disease for more than six months, partial or complete response) were rechallenged within 28 days of documented progression with ipilimumab, showing a 50% response rate [114]. This pattern of delayed response is peculiar of anti-CTLA4 antibodies and is the reason why novel immune-related response criteria were developed, according to which progressive disease is defined as an increase ≥25% in the sum of tumor diameters confirmed by two scans at least 4 weeks apart. However, anti-CTLA4 agents also exhibit a severe profile of adverse events including severe rash, grade 3-4 enterocolitis, hypophysitis, hepatitis, and more rarely, uveitis, pancreatitis, neuropathy, severe leucopenia, and red cell aplasia which are generally manageable and reversible if recognized early and treated promptly with corticosteroids [114]. Also ipilimumab produced encouraging results in phase I trails for patients with hormone-refractory prostate cancer, ovarian cancer, and non-Hodgkin's lymphoma and in phase II study of patients with metastatic clear cell renal carcinoma [114, 116]. It also significantly increased progression-free survival after conventional chemotherapy with carboplatin and paclitaxel in patients with untreated lung cancer [117]. On the basis of these data, anti-CTLA4 monoclonal antibodies represent one promising strategy to support and enhance the patient's natural antitumor response.

### 3.4. Antiangiogenic mAbs

Antiangiogenic drugs are biological therapies that stop tumors from creating their own blood vessels. There are different types of drugs that block blood vessel growth, including drugs that prevent growth factors from reaching cancer cells, drugs that block the growth factor inside the cell, and drugs that affect signals between cells. Vascular endothelial growth factor (VEGF) is one of the main proteins involved in angiogenesis [118, 119].

Bevacizumab, by blocking VEGF, can stop the receptors from sending signals necessary for blood-vessel growing. Once a receptor on a cell surface has been triggered and the pathway inside the cell activated, only tyrosine kinase inhibitors (TKIs), such as Sunitinib, can block signals that trigger the growth of new blood vessels. 

Thalidomide is another antiangiogenic drug; even if its mechanism of action is still not well known, it seems to interfere with growth signals among cells. It is helpful for refractory multiple myeloma.

### 3.5. Conjugated mAbs

As discussed above, mAbs may carry other drugs or radiation directly to cancer cells. Monoclonal antibodies can be conjugated with anticancer drugs, radioisotopes, other biologic response modifiers, or other toxins. When the antibodies bind with antigen-bearing cells, they deliver their load of drug directly to the tumour. Tositumomab and Ibritumomab are two new promising monoclonal antibodies, conjugated with radioisotopes, targeting CD20, that are still under investigation.

Antibody directed enzyme prodrug therapy (ADEPT) is a selective way for carrying an anticancer drug directly to cancer cells. The treatment is given in 2 steps. First, a mAb provided with an enzyme attached to it is administered; then, an inactive anticancer drug called a prodrug is given. When the prodrug and the enzymes meet in the cancer cell, the pro drug becomes active. This approach is still under investigation [120].

## 4. Conclusions

While the drugs reported above have clearly shown antitumor activity (a summary of some of the most promising is reported in Table 1), it is still possible to use these drugs in combination with TAA-based vaccines, cytokines, and chemotherapy.

While there are numerous immunotherapies with potential for destruction of human cancers, we are tempted to speculate that future goal of the field may be in a combination of techniques.

## Figures and Tables

**Table 1:** Summary of some of the most promising drugs currently under investigation, with their target molecule and more promising diseases of application.

Class of products	Drug name	Target	Malignancies showing promising results
Monoclonal antibodies (mAbs)	*Ipilimumab * *Tremelimumab*	CTLA-4	Melanoma*, Non-Hodgkin's lymphoma, prostate cancer, renal cell cancer
	*Rituximab*	CD20	B-cell lymphoproliferative malignances
	*Alemtuzumab*	CD52	B-CLL
	*Trastuzumab*	HER2/neu	Breast cancer
	*Cetuximab * *Panitumumab*	EGFR	CRC, head and neck cancer, and others
	*Bevacizumab *	VEGF	CRC, metastatic breast cancer, NSCLC, advanced/metastatic renal cell carcinoma
Conjugated mAbs	*Tositumomab * *Ibritumomab*	CD20	B-cell lymphoproliferative malignances
Oncogene inhibitors	*Plexxikon*	BRAF	Melanoma
Vaccines	*Sipuleucel-T*	APC presenting prostatic antigens	Prostate cancer
	*TroVax*	APC presenting 5T4 epitope	Advanced CRC, renal cell carcinoma, prostate cancer
HSP90 inhibitors	*17-AAG * *geldanamycin*	HSP90	Various cancer

Abbreviations used in the table:

APC: antigen presenting Cell,

B-CLL: B-cell chronic lymphocytic leukemia,

CRC: coloRectal carcinoma,

EGFR: epidermal growth factor Receptor,

HCC: hepatocellular carcinoma,

HSP90: heat shock protein 90,

NSCLC:* non-small-cell lung carcinoma,*

VEGF: vascular epidermal growth factor,

*This agent as most of the others may also be used in combination with TAA-based vaccines, cytokines, and chemotherapy.
